# The Microenvironment in Focus for Male Fertility

**DOI:** 10.1093/function/zqaf027

**Published:** 2025-06-23

**Authors:** Helle Praetorius

**Affiliations:** Department of Biomedicine, Aarhus University Hoegh Goldbergs Gade 10 (build 1115) 8000 Aarhus C, Denmark

## A Perspective on “Role of Pannexin 1, P2X7, and CFTR in ATP Release and Autocrine Signaling by Principal Cells of the Epididymis”

All systemic and hormonal regulation occurs on the backdrop of local autocrine and paracrine signaling. In general, the local signaling mechanism constitutes a network of simple signaling molecules released from the cells when they are stressed by outside stimuli and performs organo-preservation and self-protection (for review, see Leipziger and Praetorius^1^). All cells are protected by a parasol of local autocrine and paracrine signaling, usually through cascades of signaling molecules acting on receptor-operated systems that secure mutual redundancy. The sheer abundance of these signaling systems is overwhelming when attempting to gain a comprehensive understanding of a given tissue’s function. However, paracrine signaling is essential to make, for instance, epithelial organs consisting of several cell types working as a syncytium. Here, small signaling molecules with a fast turnover rate are exceedingly crucial for communication and a coordinated function of the tissue. ATP is a prominent example with characteristic regulated release from almost all mammalian cells, distinct downstream activation of P2 receptors, and fast degradation of membrane-associated ectoATPases (for an overview, see Praetorius and Leipziger^2^).

These deliberations on the microenvironment composition for tissue function are exceedingly relevant in reproduction, with an increased focus on live-style factors for male reproduction, even during in vitro fertilization.^3^^,^^4^ Hence, functional studies of the healthy male reproductive systems are in dire demand. Brochu et al. bring this area to the forefront of the study and provide new insights into the interplay amongst cell types in the mouse epididymis.^5^ In terms of the microenvironment, the epididymis is of particular interest, as its primary function is to establish and maintain optimal conditions for the maturation and storage of spermatozoa. The special microenvironment in the epididymis ensures that the spermatozoa remain immotile and dormant, with minimal energy consumption.^6^ This requires a low-pH, high-K^+^, and low-Na^+^ content in a high osmolality^6^ ensured via an orchestrated functional unity of the principal cells, the clear cells, and the basal cells of the epididymis. Failure to sustain this microenvironment results in premature activation of the spermatozoa and reduces the chance of spermatozoa to reach the oocyte in the female reproductive tract. The group has previously demonstrated that ATP, as a signaling molecule, ensures sufficient luminal acidity in the epididymis by stimulating the insertion of V-ATPases into the apical membrane of the clear cells.^7^^,^^8^ The signal for acid secretion is communicated from the principal cells, which have previously been shown to release ATP in a regulated fashion into the apical compartment of the epididymis.^9^ In this study, the authors demonstrate through fetching immunolocalization that ATP is released to the luminal membrane through activation of the ATP-permeable pannexin 1 channel in collaboration with the P2 × 7 channel in both the immortalized epididymal Principal cell (PC) cell line and in vivo perfused mouse epididymis. Despite the mouse epididymis expressing a variety of P2 receptors (P2Y_1,2,4,6,12,13,14_ and P2 × 2, 3, 4, and 7), the dominant functional effect of luminal ATP is mediated by P2 × 7 receptors.

Intriguingly, the authors have also previously shown that the principal cells contribute to epididymal H^+^ secretion through the apical Na^+^-H^+^ exchanger 3 (NHE_3_).^10^^,^^11^ However, this study convincingly emphasizes that the in vivo application of ATPγS induces the internalization of apical NHE_3_ in PCs. Collectively, this suggests that luminal ATP release from the principal cells stimulates acid secretion via the insertion of V-ATPases while reducing the NHE_3_ in the apical membrane of the principal cells. Although both mechanisms support acid secretion to the apical compartment, NHEs are generally believed to be able to work in reverse mode, depending on the relative ion concentrations.^12^^,^^13^ Hence, the combination of low luminal Na^+^ concentration during extensive clear cell-mediated H^+^ secretion might put principal cells in danger of intracellular acid load and might also facilitate Na^+^ secretion for a less favorable environment for the spermatozoa. At this stage, these are mere speculations far from being grounded in experimental evidence. However, the interesting findings in this study emphasize the importance of continuously hunting for a better functional understanding of the microenvironmental impact on male fertility, and the study by Brochu et al. provides a solid platform for further excavations.
